# Intravascular Lithotripsy for Calcified Left Main Artery Disease

**DOI:** 10.1016/j.jscai.2023.101126

**Published:** 2023-08-25

**Authors:** Michael S. Lee, Dean J. Kereiakes, Richard A. Shlofmitz, Evan Shlofmitz, Gary S. Mintz, Ziad A. Ali, Duk-Woo Park, Seung-Jung Park

**Affiliations:** a Innovative Medical Solutions, Los Angeles, California; b The Carl and Edyth Lindner Research Center at The Christ Hospital, Cincinnati, Ohio; c St. Francis Hospital & Heart Center, Roslyn, New York; d Cardiovascular Research Foundation, New York, New York; e New York Institute of Technology, Glen Head, New York; f Asan Medical Center, University of Ulsan College of Medicine, Seoul, South Korea

**Keywords:** coronary artery calcification, intravascular lithotripsy, left main artery

## Abstract

Left main coronary artery disease subtends a large area of potentially jeopardized myocardium. Percutaneous coronary intervention for severe left main coronary artery disease is a reasonable treatment option for select patients. Severe coronary artery calcium of the left main artery increases the complexity of percutaneous coronary intervention and is associated with increased risk of periprocedural complications and worse long-term clinical outcomes. Intravascular lithotripsy (IVL) utilizes sonic pressure waves to modify severe coronary artery calcium and has emerged as a safe and effective alternative to coronary atherectomy. However, left main lesions were excluded from regulatory approval clinical trials of IVL. Herein, we review all available data regarding the use of IVL treatment for severe left main coronary artery disease.

## Highlights

• PCI for severe LMCA disease is a reasonable treatment option for select patients.
• Severe CAC of the LMCA increases the complexity of PCI and is associated with increased risk of periprocedural complications and worse long-term clinical outcomes.
• IVL utilizes sonic pressure waves to modify severe CAC and has emerged as a safe and effective alternative to coronary atherectomy.

## Central Illustration

**Figure:**
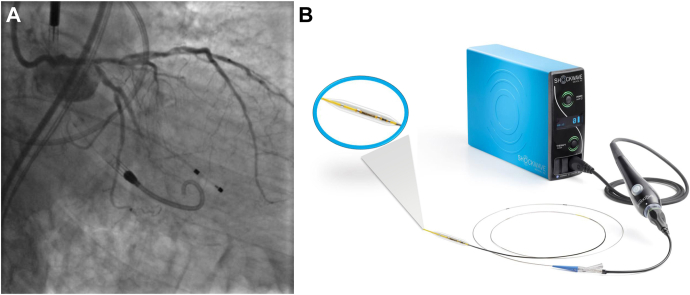

Percutaneous coronary intervention (PCI) is a reasonable treatment strategy for selected patients with severe left main coronary artery disease, especially in patients who are not suitable candidates for coronary artery bypass surgery.^1^, ^2^, ^3^ Severe coronary artery calcification (CAC) is associated with increased risk of adverse cardiac events after PCI.^4^ CAC increases the complexity of PCI as it may limit stent expansion and thus increase the risk of in-stent restenosis and stent thrombosis. Stent thrombosis of the left main coronary artery is likely to be catastrophic as it is universally associated with a large myocardial infarction, cardiogenic shock, or death given the large territory that the left main artery subtends. Severe CAC of the left main artery represents a complex lesion subset with increased rates of periprocedural complications and target vessel failure after PCI.

Appropriate plaque modification of severe CAC is crucial to maximize full stent expansion. Coronary atherectomy is a well-established treatment for severe CAC. We have shown previously that rotational and orbital atherectomy are safe and effective treatments for severe CAC of the left main artery.^5^^,^^6^

Intravascular lithotripsy (IVL) has been a “disruptive technology” in the field of interventional cardiology. Unlike coronary atherectomy, the IVL catheter is based on a semicompliant rapid exchange coronary balloon and advanced over any 0.014” guide wire. IVL modifies severely calcified plaque by circumferentially delivering pulsative sonic pressure waves from 2-wave emitters on the shaft of the balloon catheter that fracture calcium deposits in the intimal and medial vascular layers.^7^, ^8^, ^9^ IVL resulted in excellent angiographic and short-term clinical outcomes.^7^^,^^8^ However, patients with unprotected left main disease were excluded from the Disrupt CAD series of trials. In this article, we present the current literature on IVL for unprotected left main coronary artery disease and the rationale for its use compared to coronary atherectomy (Central Illustration).

**Central Illustration:**
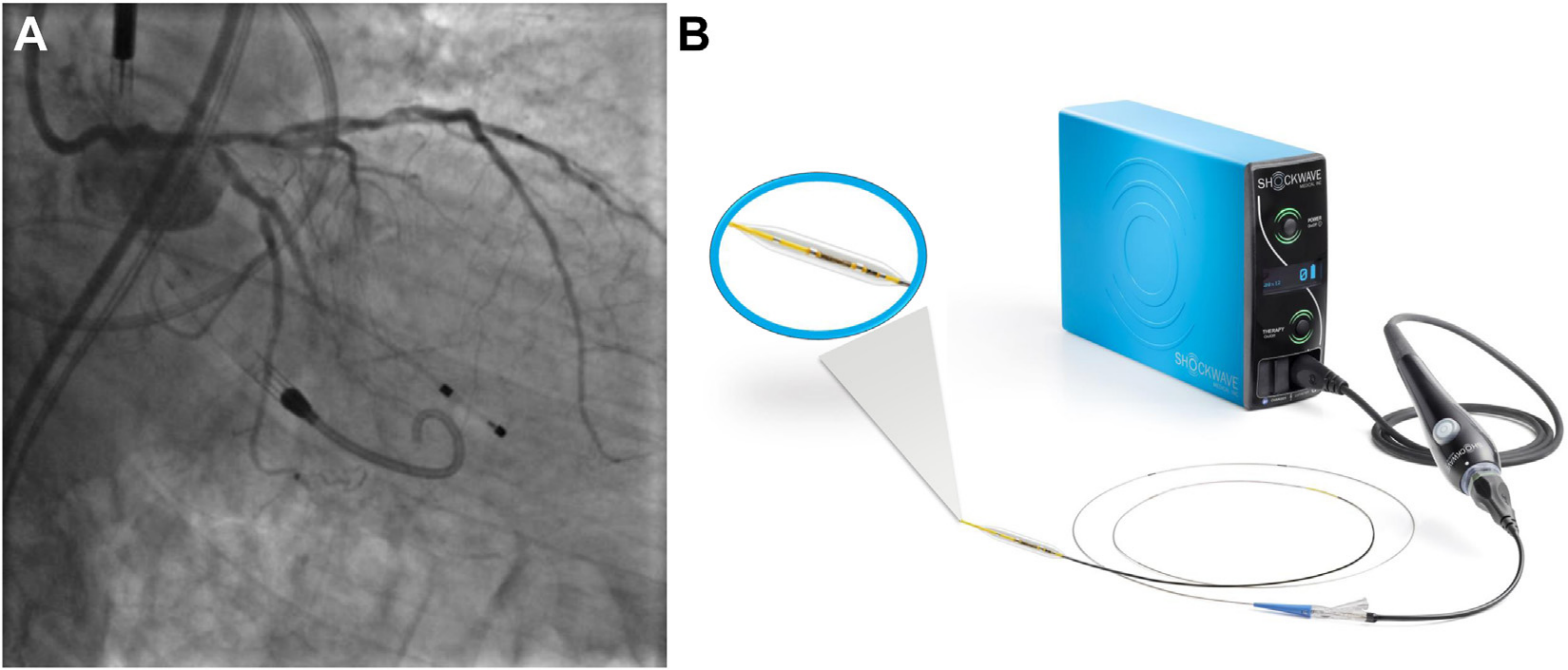
(**A**) Coronary angiography shows a severe distal left main artery lesion. (**B**) Coronary intravascular lithotripsy system with balloon catheter containing 2 emitters.

## Methodology

A search of PubMed (https://pubmed.ncbi.nlm.nih.gov) using the keywords “left main intravascular lithotripsy” yielded 29 studies from June 2019 to May 2023. Of these, we included 15 studies that reported on patients with severe CAC involving the left main artery who underwent IVL prior to PCI. Studies that included patients who underwent IVL and concomitant orbital atherectomy (“orbital-tripsy”) or concomitant rotational atherectomy (“rota-tripsy”) were excluded from the analysis.

This research was a review of published literature. Therefore, informed consent was not obtained. The coronary angiograms used for illustrative purposes in this paper were anonymized to protect the identities of the patients involved.

## Summary of existing literature

Data on IVL of the left main artery come largely from small series or case reports with limited duration of follow-up (Table 1).^10^, ^11^, ^12^, ^13^, ^14^, ^15^, ^16^, ^17^, ^18^, ^19^, ^20^, ^21^, ^22^, ^23^, ^24^ In a single-center study from the Northeast of England, 44 patients underwent PCI with IVL for unprotected left main coronary artery stenosis.^10^ IVL was used as a second step and/or bailout technique in 86.4%. Acute procedural success, defined by stent delivery with <50% residual stenosis and without serious clinical complications, was 86.4%, and the acute angiographic success rate was 88.6%. Two patients (4.5%) experienced dissection, 2 patients (4.5%) experienced perforation, and 1 patient (2.3%) experienced abrupt vessel closure. At 1 year, major adverse cardiac events (MACE) occurred in 10 of the 44 patients (22.7%). The 1-year rate of death was 9.1%, as was the rate of myocardial infarction. Ischemia-driven repeat revascularization was 7.5%.

**Table 1:** Studies of IVL of the left main coronary artery.

Study	No. of patients	Duration of follow-up	Outcomes	MCS	Intravascular imaging
Hesse et al^10^	44	1 y	MACE: 22.7%	0%	IVUS: 77.3%, OCT: 11.4%
Cosgrove et al^11^	31	30 d	MI: 3.2%	Impella: 6.5%	OCT: 100%
Salazar et al^12^	23^a^	30 d	No MACE	17.4%	83%
Rola et al^13^	15	6 mo	MACE: 13.3%	NA	20%
Rao et al^14^	15	In hospital	No cardiac death, Q-wave MI	NA	NA
Ocaranza-Sánchez et al^15^	8	1 y	MACE: 12.5%	0%	IVUS: 87.5%
Wong et al^16^	3	30 d	No MACE	0%	NA
Marchese et al^17^	2^b^	4 and 6 mo	No MACE	100%	IVUS: 50%
Çimci et al^18^	1	3 mo	No MACE	100%	0%
Ristalli et al^19^	1	In hospital	No MACE	100%	0%
Azzalini et al^20^	1	In hospital	No MACE	100%	0%
Gozolits et al^21^	1	In hospital	No MACE	0%	0%
Agrawal et al^22^	1	3 mo	No MACE	100%	IVUS: 100%
Hofmann et al^23^	1	30 d	No MACE	0%	IVUS: 100%
Kosowski et al^24^	1	In hospital	No MACE	0%	IVUS: 100%

IVUS, intravascular ultrasound; MACE, major adverse cardiac event; MCS, mechanical circulatory support; MI, myocardial infarction; NA, not available; OCT, optical coherence tomography; TLR, target lesion revascularization.

a 19 patients had unprotected left main coronary artery disease.

b Concomitant transcatheter aortic valve replacement.

Cosgrove et al^11^ reported on 31 patients who underwent PCI with IVL of left main artery disease. The distal bifurcation was involved in 23 of the 31 (74.2%) of cases. The Culotte technique was the only 2-stent strategy used to treat distal bifurcation and was used in 11 (35%) patients. Procedural success was achieved in 97%. There were no angiographic complications or in-hospital MACE, which was the composite of death, myocardial infarction, and target vessel revascularization. At 30 days, 1 patient (3.2%) experienced a non–ST-elevation myocardial infarction (NSTEMI) due to plaque rupture in a nontarget vessel (right coronary artery).

In an observational registry of 23 patients who underwent IVL for severely calcified left main disease from 6 European centers, 19 (82.6%) patients had unprotected left main disease.^12^ The majority of patients (91%) had distal bifurcation disease. No patients experienced angiographic complications including dissection, perforation, or slow flow. At 30-day follow-up, no patient experienced death, myocardial infarction, or target vessel reintervention.

In a study of 15 patients with a mean SYNTAX score of 23, the in-hospital and 6-month MACE rates were 6.7% and 13.3%, respectively.^13^ No patient experienced an angiographic complication. A fatal subacute stent thrombosis occurred 5 days after PCI. Another patient with severe left ventricular systolic dysfunction (ejection fraction 25%) died unexpectedly 14 days after discharge. When compared to 29 patients (mean SYNTAX score of 28) who underwent rotational atherectomy, there were no significant differences in in-hospital and 6-month MACE (10.3% and 17.2%, respectively).

In a study of 29 patients who underwent IVL, 15 patients had left main artery disease. Provisional single-stent strategy was performed in 12 out of 15 (80%) patients.^14^ All patients had successful stent delivery. No patients experienced in-hospital cardiac death, Q-wave myocardial infarction, target vessel revascularization, perforation, abrupt vessel closure, slow flow/no reflow, or stent thrombosis.

Ocaranza-Sánchez et al^15^ reported on 8 patients who underwent PCI with IVL of the left main coronary artery. The distal bifurcation was involved in 6 out of the 8 patients (75%). The procedural success rate was 100%. At 1-year follow-up, 1 (12.5%) patient experienced a MACE attributed to myocardial infarction and target lesion revascularization.

Wong et al^16^ reported on 3 patients, including a patient with severe ischemic cardiomyopathy and a 96-year-old who presented with NSTEMI who underwent PCI with IVL of the distal left main artery. Angiographic success was achieved in all 3 patients. No patients experienced a MACE at 30 days.

Marchese et al^17^ reported 2 high-risk patients who underwent successful PCI with IVL of the left main artery with veno-arterial extracorporeal membrane oxygenation. Angiographic success was achieved in both patients. Neither patient experienced a MACE at 4- and 6-month follow-up.

A total of 7 case reports of one patient each were found.^18^, ^19^, ^20^, ^21^, ^22^, ^23^, ^24^ At a duration of follow-up ranging from in-hospital to 3 months, no patient experienced a MACE. It included very high-risk patients including a patient with severe left ventricular systolic dysfunction (ejection fraction of 20%) and moderate to severe mitral regurgitation who underwent successful PCI of the distal left main artery with IVL with Impella support.^19^ Agrawal et al^22^ reported on a patient with NSTEMI and cardiogenic shock due to in-stent restenosis involving left main artery bifurcation who underwent successful IVL and mechanical circulatory support with Impella. The patient was free from MACE at 3-month follow-up. The ejection fraction improved from 38% prior to PCI to 55% after PCI.

Cumulative data from the 15 case series and reports revealed angiographic complications occurred in 3.4% (5/148), which were all from the English registry^10^ (Supplemental Table S1). In-hospital MACE were 1.4% (2/104) due to 2 patient deaths (1.4%). Among the 52 patients who had 1-year follow-ups, the MACE rate was 21.2%, mortality rate was 7.7%, myocardial infarction rate was 9.6%, and target lesion revascularization was 7.7%.

## Discussion

Intravascular lithotripsy appears to be a reasonable and feasible approach with preliminary safety for clinically significant left main artery disease with severe CAC. IVL was associated with high procedural success rates. Compared to coronary atherectomy, IVL has several potential advantages (Table 2). IVL is easier to use with a short learning curve, and the time to set up is shorter compared to coronary atherectomy. Unlike rotational atherectomy, which requires the RotaWire, and orbital atherectomy, which requires the ViperWire, IVL is performed with a standard workhorse wire. This helps the case proceed more expeditiously because the specialty wires are, at times, difficult to torque and traverse the lesion and often require a workhorse wire to initially traverse the lesion followed by swapping out for a specialty wire.

**Table 2:** Comparative utility of IVL and atherectomy in calcified left main coronary artery disease.

	Intravascular lithotripsy	Coronary atherectomy
Ease of use	+++	+
Time to set up	+	++
Risk of dissection	+	++
Risk of perforation	+	++
Risk of slow flow/no reflow	+	++
Use workhorse wire	+++	−a
Treatment of eccentric CAC	++	+
Maintain wire in side branch	+++	−a
Crossability	+	+++

CAC, coronary artery calcification.

a Not feasible

Intravascular imaging with either optical coherence tomography or intravascular ultrasound (IVUS) is an essential component of PCI of the left main artery. The inability of an optical coherence tomography or IVUS catheter to cross the left main artery suggests the presence of a severely stenotic lesion. It is particularly important to perform intravascular imaging prior to PCI of the left main artery to determine the reference vessel diameter, the minimum lumen area, plaque morphology, calcium distribution and severity, and the need for plaque modification of severe CAC. Intravascular imaging also facilitates the choice of plaque modification. While coronary atherectomy and IVL are effective for concentric CAC, IVL may be the preferred device for eccentric CAC. Eccentric CAC, which is usually associated with suboptimal results, may be difficult to ablate with atherectomy due to guide wire bias away from the target lesion.^25^^,^^26^ However, the IVL balloon makes circumferential contact with the vessel wall and is not impacted by guide wire bias. Intravascular imaging can help determine whether there is favorable or unfavorable guide wire bias. Calcification of the left main coronary artery, especially the distal segment, is commonly nodular.^27^ Calcified nodules are more prevalent in areas of hinge movement of the coronary such as the ostial and mid right coronary artery and in areas of high lipid burden and necrotic core, such as the left main coronary bifurcation.^27^^,^^28^ Rotational or orbital atherectomy has been favored in published algorithms for plaque modification of nodules.^29^ Theoretically the rotational, or perhaps even more favorably, orbital motion may debulk the nodule facilitating device delivery. Unfortunately, evidence to support the efficacy of these devices is limited.^30^, ^31^, ^32^ On the contrary, a recent large dataset specifically comparing the utility of calcium lesion preparation in calcified nodules to nonnodular calcium showed that IVL was a feasible frontline tool for severe coronary artery calcium with nodular morphology.^33^ The IVL catheter crossed the lesion, delivered therapy, and facilitated delivery of stents in all cases. Moreover, consistent with the pooled data from the Disrupt CAD trials, IVL was very safe for treatment of calcified nodules.^34^ Critically, there was no difference in residual area stenosis, stent area, stent expansion, or acute gain comparing calcified nodules and nonnodular calcium acutely after PCI. These data support the utility of IVL, even when the morphology of the left main lesion is nodular. Intravascular imaging performed after IVL commonly demonstrates calcium fractures. In the RENOVATE-COMPLEX-PCI trial, among patients with complex coronary artery lesions (unprotected left main coronary artery disease in 11.7% and severely calcified lesions in 14.1%), intravascular imaging-guided PCI led to a lower risk of a composite of death from cardiac causes, target vessel-related myocardial infarction, or clinically driven target vessel revascularization than angiography-guided PCI at a median follow-up of 2.1 years.^35^ Similarly, the British Cardiovascular Intervention Society registry demonstrated that IVUS-guided left main artery PCI reduced 1-year mortality.^36^

There are several procedural considerations when using IVL in unprotected left main disease. IVL may be the preferred plaque modification technique in patients with left main disease and severe left ventricular systolic dysfunction as these patients may not tolerate coronary atherectomy given the potential for myocardial depression and hemodynamic compromise from distal embolization of atheromatous debris and so-flow/no reflow phenomena. While the duration of each run of coronary atherectomy is usually 20 seconds, it is the discretion of the operator to use shorter runs to minimize the risk of ischemia and hemodynamic collapse. In contrast, IVL has not been associated with distal embolization. Patients may develop global ischemia and hemodynamic instability during prolonged IVL balloon inflation in the left main artery, particularly in patients with severe left ventricular systolic dysfunction. The shorter duration of IVL balloon inflation (15 seconds) decreases the risk of developing ischemia and is usually well tolerated. If the patient develops ischemia and hemodynamic perturbation after the IVL balloon is deflated, it may require withdrawing the balloon from the left main artery into the guiding catheter to allow perfusion until the patient achieves hemodynamic stability. The development of ischemic changes on electrocardiography should be closely monitored during IVL inflation. The decision to use a mechanical circulatory support device in these patients during IVL of the left main artery to better tolerate ischemic time should be based upon clinical judgment, including the complexity of the left main artery (distal bifurcation disease), the presence of multivessel coronary artery disease, chronic total occlusion of the right coronary artery, which is collateralized by the left coronary system, and cardiac hemodynamics (especially cardiac power output and left ventricular end-diastolic pressure) (Table 3).

**Table 3:** Considerations during intravascular lithotripsy of the left main artery.

Left ventricular systolic dysfunction	Consider mechanical circulatory support device if severe left ventricular systolic dysfunction, complex coronary anatomy, poor baseline hemodynamics. Shorten the duration of IVL inflation to minimize the risk of ischemia.
Level of anticoagulation	Standard dosing for heparin to achieve activated clotting time of 250-300 s for Hemotech or 300-350 s for Hemochron.
Intravascular imaging	Strongly recommended pre-IVL to determine size, plaque and distribution, and post-IVL to confirm plaque modification and improvement in minimum lumen area.
Guide extension catheter	Facilitate IVL delivery in challenging anatomic lesions.

IVL, intravascular lithotripsy.

**Figure 1:**
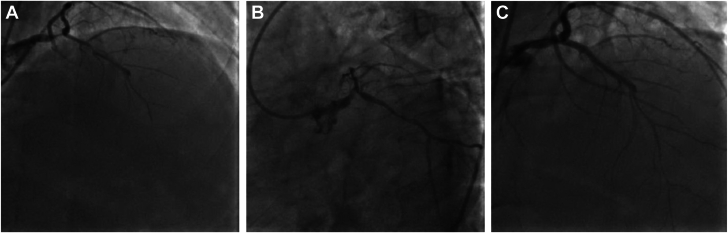
(**A**,**B**) Coronary angiography demonstrates severely calcified distal left main coronary artery bifurcation disease in the LAO-caudal projection and severe, diffuse disease involving the left anterior descending artery in the AP-cranial projection. (**C**) Final angiography in the AP-Cranial projection demonstrates satisfactory stent expansion after intravascular lithotripsy of the distal left main coronary artery followed by PCI with the double-kiss crush technique. LAO, left anterior oblique; PCI, percutaneous coronary intervention.

Distal bifurcation disease of the left main artery is common (70%).^37^ Severe CAC can involve both the ostial left anterior descending (LAD) and left circumflex (LCX) arteries (Medina 1, 1, 1) and may require plaque modification of both vessels (Figure 1). In a subgroup analysis of patients with bifurcation disease in the PREPARE-CALC trial, lesion preparation with rotational atherectomy resulted in fewer instances of side-branch compromise compared with scoring/cutting balloons.^38^ If the ostial LAD is treated first with atherectomy, the wire in the LAD needs to be removed prior to performing atherectomy of the LCX. There is a risk that the wire might not recross into the LAD. However, the guide wire position can be maintained in both the LAD and LCX during IVL, without the loss of wire position. Although case reports of branch-vessel rotational atherectomy while maintaining the side-branch wire “protected” with a microcatheter have been described, the microcatheter can be abraded/scored by the atherectomy burr, and catheter fragments can be embolized.

Treatment of in-stent restenosis of previously implanted left main stents that were suboptimally expanded due to severe CAC poses a clinical dilemma. Coronary atherectomy represents a poor option as the burr or crown is unlikely to significantly modify the calcified plaque behind the stent struts. Coronary atherectomy can also lead to disruption of the stent struts and distal embolization of stent debris. IVL represents a reasonable treatment option for in-stent restenosis due to severe CAC as the sonic pressure waves can traverse the stent struts and modify the calcified plaque without disrupting the integrity of the stent struts. However, the use of IVL has no formal indication for the treatment of in-stent restenosis, and its use is considered off-label. Furthermore, there are bench test concerns regarding polymer disruption when IVL is performed early after implantation of drug-eluting stents.^39^

A potential advantage of IVL is the relatively low incidence of periprocedural complications. Unlike atherectomy, in which a rotational atherectomy burr and orbital atherectomy crown rotate at high speeds that may perforate or dissect the vessel, the IVL balloon is inflated at low pressure (4 atm), which mitigates barotrauma to the vessel and may provide a safety advantage. IVL’s safety advantage over coronary atherectomy may be more pronounced in tortuous and angulated anatomy.^40^ The LCX can come off the left main artery at an acute angle, which increases the risk of extreme wire bias and “guttering” with coronary atherectomy and angiographic complications like dissection.

If rotational atherectomy of the left main artery is performed, a 1.75-mm burr, or larger, may be required. Larger burrs require at least a 7F guide catheter, which occasionally may render transradial intervention unfeasible. The 4-mm diameter IVL balloon is compatible with a 6F guide catheter, which allows the operator to perform transradial intervention.

A disadvantage of IVL is that the balloon is bulky with a crossing profile of 0.043” to 0.046”, which may not traverse severely stenotic lesions. A very low-profile balloon can be used to predilate the severely stenotic lesion in order to deliver the IVL balloon. The use of a guide extension catheter can facilitate IVL delivery not only in severely stenotic lesions but also in tortuous and angulated anatomy. However, coronary atherectomy using a small (1.25 or 1.5 mm) burr or crown may be the preferred strategy for uncrossable lesions despite these maneuvers. Subsequent IVL can be performed at the discretion of the operator.

In a retrospective study of 54 patients who underwent coronary IVL, the incidence of coronary IVL-provoked ventricular capture was 77.8% (n = 42).^41^ Ventricular capture was associated with a fall in systolic blood pressure of between 10 and 35 mm Hg that immediately resolved on return of intrinsic rhythm. However, no adverse clinical events occurred among patients in whom coronary IVL-provoked ventricular capture. Of the 42 patients who had IVL-provoked ventricular capture, 7.1% (n = 3) had IVL of the left main artery. Of the 12 patients who did not have ventricular capture, 25% (n = 3) had IVL of the main artery.

There is a paucity of prospective data on calcium modification in left main artery disease. The only study registered on clinicaltrials.gov is the IVL Left Main study (NCT04319666), which is a prospective nonrandomized pilot study to investigate the mechanical and procedural outcomes and safety of distal left main artery stenting with coronary IVL in addition to standard techniques in patients with calcific left main disease and a clinical indication for revascularization.^42^

## Conclusion

Severe CAC of the left main artery increases the complexity of PCI and is associated with worse clinical outcomes. There are limited data with IVL of the left main artery with no prospective, randomized clinical trials, or long-term follow-up. However, coronary IVL for severe CAC of the left main artery appears to be a reasonable and feasible approach for plaque modification, especially given its ease of use compared to coronary atherectomy. Intravascular imaging prior to PCI to determine the severity and distribution of CAC is highly recommended. Dedicated trials with long-term follow-up comparing different plaque modification technology are needed to establish the preferred revascularization strategy for severely calcified left main artery disease.
